# Structural Basis for Self-Discrimination by Neoantigen-Specific TCRs

**DOI:** 10.21203/rs.3.rs-2531184/v1

**Published:** 2023-01-31

**Authors:** John P. Finnigan, Jenna H. Newman, Yury Patskovsky, Larysa Patskovska, Andrew S. Ishizuka, Geoffrey M. Lynn, Robert A. Seder, Michelle Krogsgaard, Nina Bhardwaj

**Affiliations:** 1 Icahn School of Medicine at Mount Sinai; One Gustave L. Levy Pl., New York, NY; 2 Tisch Cancer Institute, Icahn School of Medicine at Mount Sinai; 1470 Madison Ave., New York, NY; 3 Department of Medicine, Division of Hematology and Medical Oncology, Mount Sinai Hospital.; 4 Brigham and Women’s Hospital, Department of Surgery, Division of Thoracic and Cardiac Surgery; 75 Francis St., Boston, MA; 5 Department of Pathology, New York University Grossman School of Medicine, New York, NY, USA; 6 Laura and Isaac Perlmutter Cancer Center at NYU Langone Health; 7 Vaccine Research Center, National Institute of Allergy and Infectious Diseases, National Institutes of Health, Bethesda, MD, USA.; 8 Vaccitech North America, Baltimore, MD, USA; 9 Parker Institute for Cancer Immunotherapy

**Keywords:** immunology, cancer, TCR, neoantigen, structure

## Abstract

Physical interactions between T cell receptors (TCRs) and mutation-derived tumour neoantigens (neoAg) presented by major histocompatibility class-I (MHC-I) enable sensitive and specific cytolysis of tumour cells. Adoptive transfer of neoAg-reactive T cells in patients is correlated with response to immunotherapy; however, the structural and cellular mechanisms of neoAg recognition remain poorly understood. We have identified multiple cognate neoAg:TCRs from B16F10, a common murine implantable tumour model of melanoma. We identified a high affinity TCR targeting H2-D^b^-restricted Hsf2_K72N_ that conferred specific recognition of B16F10 *in vitro* and *in vivo*. Structural characterization of the peptide-MHC (pMHC) binary and pMHC:TCR ternary complexes yielded high-resolution crystal structures, revealing the formation of a solvent-exposed hydrophobic arch in H2-D^b^ that enables multiple intermolecular contacts between pMHC and the TCR. These features of structural stability strikingly mimic that of a previously published influenza peptide-H2-D^b^ complex and its corresponding TCR, suggesting that there are shared structural motifs between neoantigens and viral peptides that explain their shared immunogenicity.

The TCR is a variable heterodimeric protein complex that non-covalently binds to the surface-bound peptide-major histocompatibility complex (pMHC), which presents peptide antigens derived from degraded intracellular proteins^1^. Anti-tumour T cell immunity is mediated by the physical interaction between T cell receptors (TCR) and tumour antigens presented by pMHC on tumour cells^2^. Tumour cells accumulate somatic non-synonymous mutations encoding variant proteins that degrade to form mutation-derived tumour neo-antigens (neoAg)^3^. Analogous to pathogens, tumours evolve in hosts under selective pressure from endogenous and treatment-induced immunity^4^. However, immunogenic neoAg can persist despite selective immunoediting and are increasingly recognised as the primary target of tumour-reactive TCRs^5–7^. There are now multiple clinical trials associating neoAg-reactive T cells with positive clinical outcomes for patients treated with therapeutic vaccines^8–12^, cell-based therapies^13–15^, and immune checkpoint blockade^16–20^. However, because of historical difficulties associated with prospectively studying clinically-relevant human neoAg-reactive TCRs, only a fraction of the TCRs identified to date have received detailed *in vitro* and *in vivo* characterization^13, 21–23^.

Studies demonstrate a causal relationship between neoAg-reactive T cells and radiographic regression of established tumours and/or prolonged disease-free and overall survival^16^. High functional avidity/structural affinity has emerged as a recurrent feature of neoAg-reactive TCRs and may be necessary to recognise tumour cells naturally selected for low target antigen surface density. In early examples, this has been shown to potentially derive from TCR recognition of structural differences between mutation-derived neoAg and the corresponding wild-type product, but the broader generalizability of these findings remains unknown^24^. Many other core questions remain unanswered, such as why non-synonymous mutations are rarely recognised by TCRs; and how some neoAg-reactive TCRs selectively recognise mutated peptides and do not cross-react with the corresponding wild-type peptides, whereas others exhibit significant cross-reactivity. Structure-guided mechanistic answers to these questions might enable the prediction of neoAg-reactive TCR activity as well as potential toxicities resulting from cross-reactivity, thus enabling the rapid translation of safe and effective neoAg-reactive TCRs into the clinic.

To systematically address these and other questions pertaining to neoAg-reactive TCRs, we employed the B16F10 murine melanoma cell line. B16F10 is an orthotopic implantable tumour model syngeneic to C57BL/6 mice that exhibits limited spontaneous immunogenicity and is refractory to multiple types of immunotherapy. We reasoned that neoAgs identified in this model would appropriately represent neoAgs in advanced human cancers, thereby improving the translational relevance of our findings. To identify expressed non-synonymous mutations, we first performed exome and transcriptomic sequencing of the B16F10 and selected a subset of these putative neoAg based on published ranking criteria for *in vivo* validation^25^. We then characterised the vaccine-induced CD8^+^ T cell response to seven novel neoAgs. Finally, using biochemical and cellular assays in combination with high-resolution crystal structures of the prototype murine neoAg Hsf2 p.K72N neoAg-MHC complex, with and without a corresponding reactive TCR, we determined the structural requirements for TCR antigen recognition and selectivity, as well as conditions for tumour cell recognition *in vitro* and tumour growth control *in vivo*.

## Identification of Model Neoantigens in the B16F10 Model

**Extended Data Fig. 1 |:**
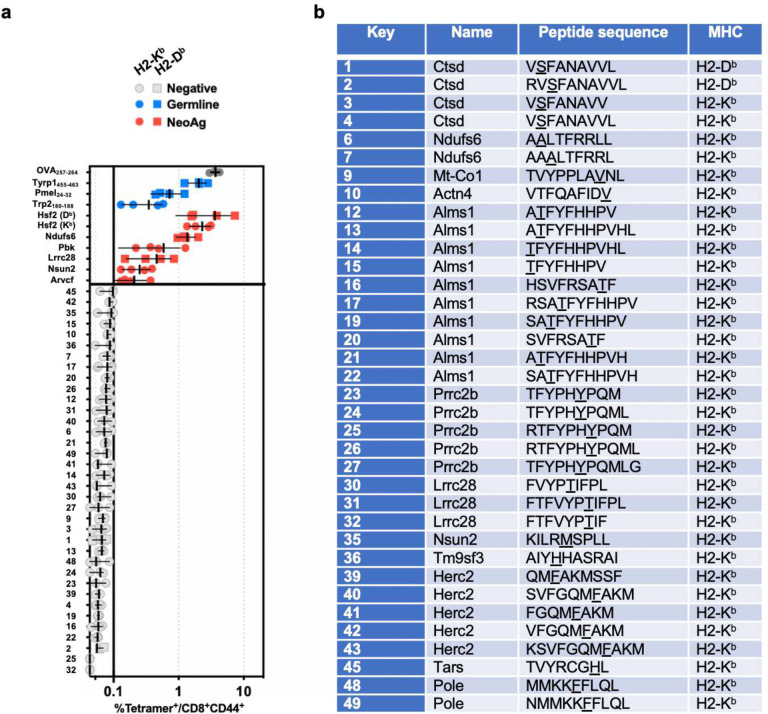
Identification of neoantigen-reactive T cells with peptide vaccination in non tumour-bearing C57BL/6 mice. **a**, C57BL/6 mice (n=4/group) were immunised with a single dose of peptide vaccine targeting putative B16F10 neoantigens (neoAgs) or non-mutated (germline) tumour antigens (listed, also see Fig. 1e). Putative neoAg epitopes that did not elicit a T cell response are labeled as “negative”. Symbol indicates the frequency of pMHC tetramer-bound CD8^+^CD44^+^ T cells in peripheral blood 7 days post-immunization. **b**, Key for negative antigens shown in a. Underlined amino acids highlight mutated amino acid uniquely present in B16F10.

**Fig. 1 |:**
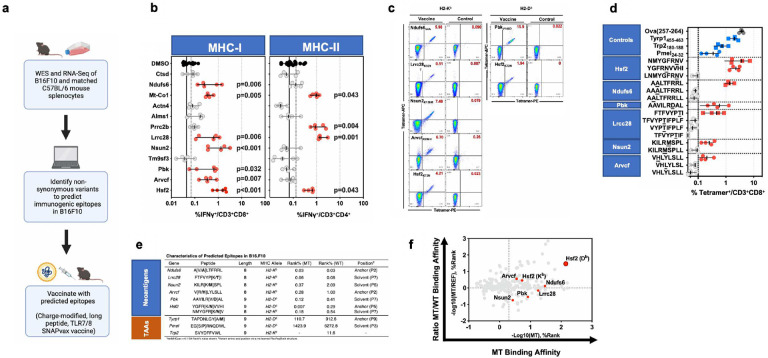
Identification of neoantigenic epitopes in the B16F10 melanoma model. **a**, Schematic depicting the workflow to identify neoantigens in B16F10. **b**, C57BL/6 mice (n=4–5/group) were immunised with a single dose of peptide vaccine targeting putative B16F10 neoantigens (listed). Seven (7) days post-immunization splenocytes were isolated and total T cells stimulated with titrated mutant peptide for 6 hours then IFN production was measured by intracellular staining (ICS) for flow cytometry. Symbol indicates individual subject (n=4–5/condition), ± 95% confidence interval (CI). Solid line indicates assay lower limit of detection (LLD). Dashed indicates upper limit of 95% CI for negative responses. Red color highlights antigens that elicited responses above the LLD and were further studied for characterization. **c**, C57BL/6 mice (n=4/group) were immunised with a single dose of peptide vaccine targeting putative B16F10 neoantigens (listed). Seven (7) days post-immunization, flow cytometry was performed on splenocyte-derived CD8^+^ T cells. Representative flow cytometry plots of tetramer staining with indicated tetramers are shown. **d**, As in **b**. Blue color highlights tumour-associated antigens (TAAs), gray color highlights non-tumour Ova antigen control. **e**, Attributes of neoantigens (top) predicted by computational algorithm and TAAs previously characterised in the literature (25–26). BA-Rank values are shown for both mutated (MT) and wild type (WT) peptides. **f**, *In silico* MHC-I binding affinity analysis of Hsf2 p.K72N_68–76_ demonstrates high affinity binding and high differential binding affinity between mutant (MT) and corresponding wild type (WT) peptides.

To identify B16F10 neoAg, we performed paired exome sequencing of cultured B16F10 murine melanoma cells and C57BL/6 splenocytes, as well as bulk RNASeq of resected B16F10 tumours (Fig. 1a). We then integrated these data and identified putative neoAg using published methods^25,26^. We then performed murine immunization studies using a peptide-based vaccine^27,28^. We observed both vaccine-elicited neoAg-specific CD4^+^ and CD8^+^ T cells amongst splenocytes (Fig. 1b,c, Extended Data Fig. 1) and then characterised the minimal peptide epitope for seven MHC-I-restricted neoAg (Fig 1d). A summary of immunogenic MHC-I-restricted epitopes, as well as four non-mutated previously characterised tumour antigens^29–31^, is shown (Fig. 1e,f).

## Neoantigen-reactive CD8^+^ T cells recognise cognate peptide *in vitro* and *in vivo*

**Extended Data Fig. 2 |:**
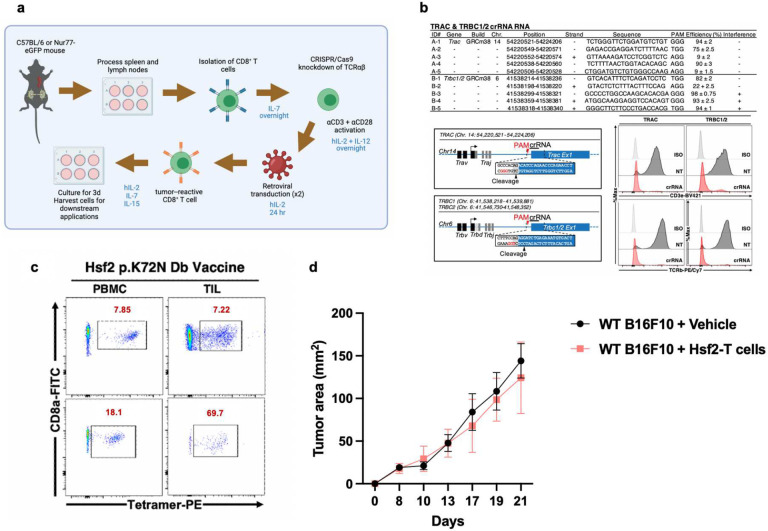
Design of engineered transgenic TCR (tgTCR) CD8^+^ T cells and *in vivo* characteristics of vaccine-elicited immunity and adoptive cell transfer. **a**, Schematic depicting process of engineering transgenic TCR (tgTCR) neoantigen or tumour associated antigen-reactive CD8^+^ T cells. hIL-2=human IL-2. **b**, Table detailing CRISPR RNA (crRNA) targeting the TRAC and TRBC loci (encoding TCRα and TCRβ, respectively) (top). Diagram of cleavage sites within TRAC and TRBC is shown (bottom, left), as well as flow cytometry staining for surface TCR expression (bottom, right), comparing isotype control-stained samples (iso), cells that were not transfected with TRAC/TRBC CRISPR/Cas9 reagents (NT), and cells that underwent CRISPR/Cas9-mediated TRAC/TRBC knockdown (crRNA). **c**, Representative flow cytometry plots of tetramer staining of T cells from peripheral blood mononuclear cells (PBMCs) or tumour-infiltrating lymphocytes (TILs) corresponding to Fig. 2f,g. **d**, C57BL/6 mice (n=3 mice/group) received 2.0×10^5^ wild type (WT) B16F10 cells (harbouring natively low expression of Hsf2 neoantigenic epitope) intradermally in the flank, were irradiated (whole body, 5 Gy) 6 days later, and administered 2.0×10^7^ 47BE7 Hsf2-reactive CD8^+^ T cells (ACT) or phosphate-buffered saline (Vehicle) intravenously (i.v.) one day later. hIL-2 was administered at 180,000 IU/mouse on day of ACT and daily for 2 subsequent days; supplementary doses were administered weekly until humane endpoint.

**Fig. 2 |:**
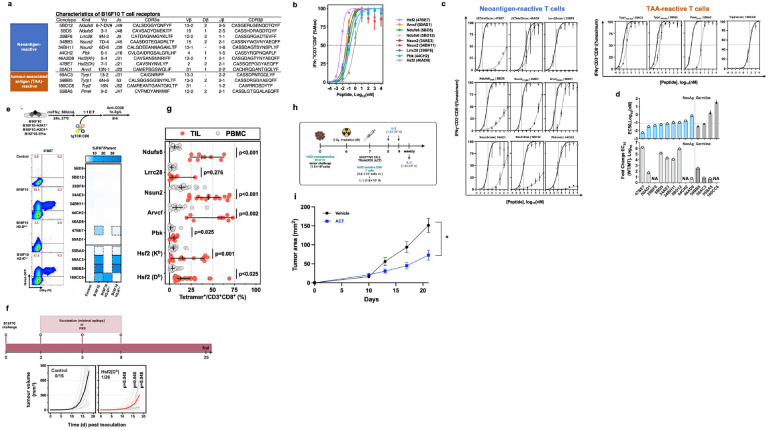
Neoantigen-reactive CD8^+^ T cells recognise cognate peptide *in vitro* and *in vivo*. **a**, Table detailing attributes of tumour-reactive T cell receptors (TCRs), including neoantigen-reactive (top) and tumour-associated antigen-reactive (TAA-reactive) (bottom) TCRs isolated upon vaccination. **b**, Neoantigen-reactive CD8^+^ T cell responses were induced by vaccination; isolated tetramer^+^ T cells were stimulated for 6 hours with (varying concentrations of) cognate peptide and αCD28 and subsequently analyzed via intracellular staining (ICS) flow cytometry for IFNγ expression. **c**, Transgenic (tg)TCR CD8^+^ T cells were co-incubated with varying concentrations of cognate mutant (MT) peptide or wild type (WT) peptide (x-axis) and αCD28 for 6 hours; intracellular staining (ICS) was performed subsequently. Percentage of T cells expressing IFNγ of a parent CD3^+^CD8^+^ population is shown on y-axis, normalised to maximum IFNγ expression. MT peptide values are shown as clear circles, and WT as filled, black circles. Trp2-reactive TCR 180CC6 only recognises a WT peptide (shown with clear circle). **d**, Neoantigen-reactive TCR half-maximal (EC_50_) cytokine production concentration (top). TgTCR CD8^+^ T cells expressing the indicated TCR were stimulated, as described in **c**, with titrated mutant (MT) or wild-type (WT) peptide and IFNγ production was measured by ICS. Symbol indicates median of biologic replicates (n=3/condition), ± 95% CI. Dashed horizontal line indicates mean half-maximal response (EC_50_) for tested neoAg TCR. (Bottom) Ratio of WT/MT EC_50_ as log_10_ fold change. **e**, Wild type B16F10, B16F10 lacking either MHC-I H2-D^b^ or H2-K^b^ (B16F10-H2D^b−/−^ and B16F10 H2K^b−/−^, respectively) or B16F10-EF1a (overexpressing neoantigenic or TAA peptide) were plated, exposed to recombinant murine IFNγ (rmIFNγ), and T cells expressing tgTCRs engineered on a Nr4a1-eGFP mouse splenocyte background were added at a 1:1 effector:target (E:T) ratio and co-incubated. Cytokine production was measured by intracellular staining (ICS) flow cytometry (n=3/condition). Representative flow cytometric analysis of CD8^+^TCR-47BE7^+^ (Hsf2-reactive) cells exposed to B16F10 target cells is shown. Nr4a1-GFP is a marker of TCR signal transduction. Numbers indicate percentage of cell population within the indicated gate. Heatmap shown summarises the frequency of CD8^+^tgTCR^+^IFγ^+^. **f**, C57BL/6 mice (n=16–26/group) were treated with Hsf2 neoantigen vaccine or PBS (mock) in accordance with timeline shown (top). tumour growth was monitored via external caliper measurement and plotted versus time since tumour inoculation (bottom). **g**, Tetramer staining on CD3^+^ CD8^+^ T cells isolated from tumour-infiltrating lymphocytes (TIL) or peripheral blood mononuclear cells (PBMCs) from mice vaccinated with each of the neoantigens listed, using same methodology as described in **b. h**, Schematic describing administration of ACT *in vivo*. C57BL/6 mice (5–10 mice/group) received 1.5×10^5^ Hsf2-overexpressing B16F10 cells intradermally in the flank, were irradiated (whole body, 5 Gy) 6 days later, and administered 2.0×10^7^ 47BE7 Hsf2-reactive CD8^+^ T cells (ACT) or phosphate-buffered saline (Vehicle) intravenously (i.v.) one day later. **i**, tumour growth was measured over time by external caliper measurement every 2–3 days after ACT or vehicle treatment and plotted using Graph Pad Prism 7. *p<0.05, two-way ANOVA with Bonferroni correction.

Next, using pMHC tetramer-based single-cell sorting and 5’ RACE PCR followed by TCR sequencing we cloned nine corresponding neoAg-reactive TCRs, as well as four non-mutated tumour antigen-specific TCRs (Fig. 2a). Then, using either vaccine-elicited or retrovirus-transduced TCR-transgenic (tgTCR) CD8^+^ T cells (Extended Data Fig. 2a,b), we confirmed antigen-induced cytokine production for all identified TCRs (Fig. 2b,c). The TCRs exhibited variable selectivity for their cognate neoAg ranging from complete specificity (29BF8, 44CH2) to complete cross-reactivity (46AD8, 50AD1) (Fig. 2d). We then assessed TCR recognition of B16F10 target cells. Notably, only TCR 47BE7, which targets the H2-D^b^-restricted neoAg Heat Shock Protein 2 (Hsf2 p.K72N_68–76_), exhibited T cell effector function *in vitro* (Fig 2e). *In vivo*, therapeutic immunization targeting Hsf2 p.K72N elicited an enrichment of 47BE7^+^ CD8^+^ T cells amongst tumour-infiltrating lymphocytes (TILs) (Extended Data Fig. 2c) and delayed tumour growth (Fig. 2f,g). Adoptive cell transfer of tgTCR 47BE7^+^CD8^+^ T cells delayed B16F10 tumour growth, contingent upon sufficient tumour expression of Hsf2 p.K72N (Fig. 2h,i, Extended Data Fig. 2d). Given the demonstrated sensitivity, selectivity, and functional activity, the Hsf2_K72N_-reactive TCR was selected for further characterization.

## Biophysical analysis of H2-D^b^/Hsf2 p.K72N_68–76_

We and others have previously shown that the position of the mutated amino acid with respect to peptide length can be used to organ neoAg into two principal classes^32–33^. Namely, neoAg in which the mutated amino acid side-chain is solvent facing and may form intermolecular bonds with incoming TCR directly (class I); versus neoAg in which the mutated amino acid side-chain is buried within the MHC-I binding pocket and therefore variably interacts with incoming TCR (class II).

**Fig. 3 |:**
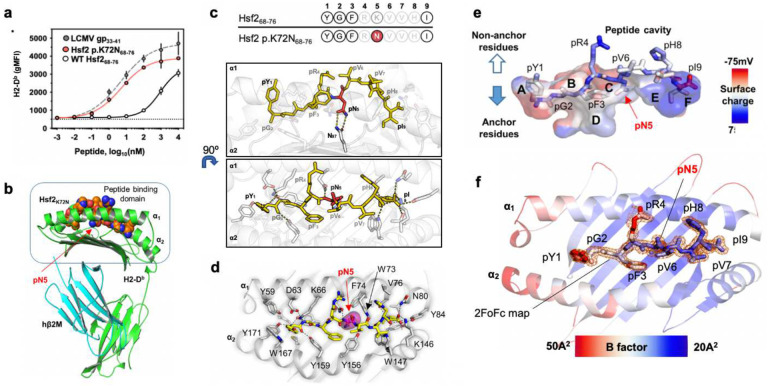
Structure of the pMHC binary complex. **a**, RMA-S cells were plated at 25°C for 18 hours, then co-incubated with the indicated peptides for 30 minutes at 30°C, followed by 3 hours at 37°C. Surface H2-D^b^ geometric mean fluorescence intensity (gMFI) was measured by flow cytometry and plotted for all peptide concentrations tested. Symbol indicates the mean of biologic replicates (n=3), ±95% confidence interval (CI). **b**, Crystal structure of H2-D^b^/Hsf2 p.K72N_68–76_. H2-D^b^ is colored in green, human β2M (hβ2M) is shown in blue. pN_5_ refers to the asparagine (N) located at peptide position 5. The peptide atoms are shown as van der Waals spheres in which oxygen is colored red, nitrogen is blue and carbon is orange. **c**, Residues participating in intermolecular contacts between H2-D^b^ and Hsf2p.K72N_68–76_. H2-D^b^ residues (white), Hsf2 p.K72N conserved residues (yellow), and mutant pN_5_ residue (red). **d**, The polar interaction network between bound epitope and H2-D^b^ Amino acid residues are presented as sticks and hydrogen bonds (distance cutoff < 3.5 Å) are shown with dotted lines. Residues are color-coded based on molecular properties; red (acidic), blue (basic), brown (aromatic), green (polar), yellow (nonpolar). **e**, Peptide-binding cavity of H2-D^b^ is shown. Hsf2 p.K72N_68–76_ residues are shown as sticks, with atoms colored according to charge. The approximate location of each binding pocket is marked by a letter from A (N-terminal pocket, residue P1) to F (C-terminal pocket, residue P9). **f**, The peptide binding domain of H2-D^b^ (cartoon) and Hsf2 p.K72N_68–76_ are colored according to the B-factor values. Peptide residues are shown as sticks. The 2FoFc map (σ cutoff 1.0, radius cutoff 1.5Å) is drawn around peptide residues only.

Hsf2 p.K72N_68–76_ is a H2-D^b^ restricted peptide (_68_YGFR**N**VVHI76) derived from Heat shock factor 2 *(Hsf2*, Uniprot: P38533). The underlying point mutation results in substitution of a basic Lys/K residue at position 5 of wild type Hsf2_68–76_ (pK_5_; ‘p’ indicating peptide residue, with the number designating the position of the residue in the peptide starting from the N-terminus) for a polar non-charged residue Asn/N (pN_5_). *In silico* binding analysis demonstrated that both Hsf2 p.K72N_68–76_ [0.007 %Rank] and Hsf2_68–76_ [0.29 %Rank] were predicted to bind H2-D^b^, but a significant affinity differential exists between the mutant (MT) and wild type (WT) peptide (Fig. 1f). Cell-based RMA-S binding confirmed this with observed half-maximal stabilization of peptide-bound H2-D^b^ (EC_50_) by Hsf2 p.K72N_68–76_ at 4.985nM [95%CI 4.21–5.92] that was comparable to control agonist peptide LCMV gp_33–41_ 4.38nM [95%CI 2.09–10.4], and significantly lower than that of Hsf2_68–76_ 883nM, [95%CI 773.9–1033] (Fig. 3a). Based on the observed binding affinity differential we hypothesised that the immunogenicity of Hsf2 p.K72N is *primarily* derived from physical interactions between the mutant residue pN5 with H2-D^b^. Thus, Hsf2 p.K72N is a protype murine class II neoAg, and, understanding the nature of these interactions may elucidate the mechanism leading to the immunogenicity of Hsf2 p.K72N_68–76_, and class II neoAg generally.

Analysis of published H2-D^b^ crystal structures demonstrates a conserved peptide binding mode mediated by *primary* hydrophobic interactions between conserved residues lining the H2-D^b^ *A-B-D* and *F-pockets* and peptide N-/C-terminal anchor residues^34^. Additionally, H2-D^b^ bound peptides form characteristic *secondary* polar interactions within the MHC-I *C-pocket*, mediated by bi-directional hydrogen bonds between asparagine residues H2-D^b^ N_97_ and peptide position 5 (pN_5_). The hydrogen bond mediated by H2-D^b^ N_97_ leads to biased presentation of peptides with pN_5_. Lastly, H2-D^b^ is defined by a conserved hydrophobic bridge formed by the side chains of W73 (α1-helix), W_147_ and Y_156_ (α2-helix) that runs perpendicular to the binding cleft and imparts an arched solvent-accessible conformation to residues in p6-p8 of H2-D^b^ bound peptides which is absent from H2-K^b^ bound peptides^34^. We hypothesised that Hsf2 p.K72N_68–76_, but not WT Hsf2_68–76_, satisfied the pN5 requirement imposed by H2-D^b^, and that the p6-p8 residues would form a solvent-exposed ridge accessible to incoming TCR. To validate this, we produced the soluble H2-D^b^/Hsf2 p.K72N_68–76_ (YGFRNVVHI) molecules as previously described, then solved the crystal structure of the binary peptide-MHC complex to 1.74A (Fig. 3b)^35,36^. We observed a typical H2-D^b^ peptide binding mode mediated by hydrophobic interactions between the H2-D^b^ A, B, D, and F-pockets and buried peptide residues pY1-pF3 and pI9, respectively—all of which are conserved between both MT/WT peptides and therefore unlikely to produce differential immunogenicity (Fig. 3c,d). The *A-pocket* is occupied by the pY1 side chain, which is solvent facing but remains buried by H2-D^b^ (Total surface area *[TSA]* 269.80 Å^2^, Buried surface area [BSA] 233.32 Å^2^), with the terminal amine engaged in hydrogen bonds with H2-D^b^ Y_7_, Y_59_, and Y_171_. The *B-* and *D-pockets* are occupied by pG2 (ASA 71.16 Å^2^, BSA 62.98 Å^2^) and pF3 (ASA 168.15Å^2^, BSA 132.91 Å^2^), both of which are also buried within the H2-D^b^ binding cleft (Fig. 3e). However, the respective side chains exhibit poor compatibility with the polar cavities, which are instead filled with nine water molecules, possibly resulting in weak binding of the peptide N-terminal segment. The *F-pocket* is fully occupied by the conserved pI9 (ASA 230.05 Å^2^, BSA 230.05 Å^2^), which is stabilised by buried hydrophobic side-chain interactions and polar interactions with the exposed terminal carboxyl group and H2-D^b^ N80 and K146. The mutated pN5 residue is buried (ASA 121.28 Å^2^, BSA 117.47 Å^2^) within the *C-pocket* and the primary amide is engaged in a hydrogen bond network with H2-D^b^ Asn_97_. This interaction is predicted to contribute binding free energy (ΔG_i_°) −0.88 kcal/mol, consistent with the behavior of an anchor residue. The remaining peptide side chains—pR_4_ and pV_6_-pV_7_-pH_8_—are solvent-exposed and do not contribute to MHC-I binding. Consistent with published studies, pV_6_ (TSA 97.07, Solvent accessible surface area [SASA] 76.41 Å^2^) and pV_7_ (TSA 96.67, SASA 22.23 Å^2^) form an arch over the W_73_-Y_147_-W_147_ bridge and projects towards A_152_ of the α2-helix, with pH_8_ projecting back towards the α2-helix. Analysis of crystallographic Debye-Waller factor/B-factors (DWF) revealed asymmetry in the distribution of peptide all-atom flexibility with N-terminal residues proximal to the *secondary* N5 anchor exhibiting increased average DFW values relative to distal C-terminal residues, pY_1_-pR_4_ [39.05±4.97] versus pN_5_ – pI_9_ [27.64±1.56] (Fig. 3f). Overall, this arrangement suggests H2-D^b^/Hsf2 p.K72N_68–76_ is stabilised primarily at the C-terminus, via the conserved pIle9 anchor *as well as* the pAsn_5_ anchor created by the p.K72N mutation with limited contribution from the N-terminal segment. Additionally, the C-terminal region’s thermal rigidity and solvent exposure suggest this segment may be preferentially targeted by H2-D^b^/Hsf2 p.K72N_68–76_ reactive TCR^34^. The combined structural and biochemical binding affinity data suggest that WT Hsf2_68–76_ with an N-terminal segment that is poorly compatible with H2-D^b^, and lacking pN5 anchor would fail to stabilise H2-D^b^, thereby explaining the differential binding affinity of the MT and WT peptides. Furthermore, the absence of the pN5 anchor would fail to stabilise the p6-p8 solvent exposed peptide ridge for interaction with incoming TCR. Finally, the fact that this feature results from the neoAg-specific pN_5_ anchor residue provides a plausible structural correlate for the published association between neoAg peptide-MHC stability in solution and CD8^+^ T cell reactivity^37^.

## Biophysical analysis of TCR-47BE7:H2-D^b^/Hsf2^pK72N^

**Extended Data Fig. 3 |:**
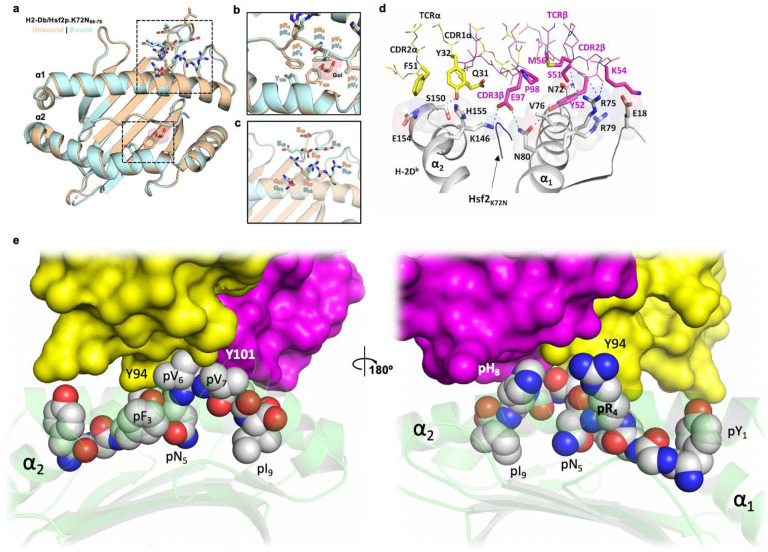
Additional structural diagrams depicting the pMHC binary complex. **a**, Bound (blue) and unbound (tan) structure of H2-D^b^/Hsf2 p.K72N_68–72_. Dotted boxes highlight p. K72N_68–72_. **b**, View of H2-D^b^ Y_156_ rotated towards D pocket in bound structure due to presence of glycerol (Gol) molecule. **c**, View of H2-D^b^ α_1_ helix solvent-exposed residues and H2-D^b^ loop A. **d**, Interface between TCR-47BE7 and H2-D^b^ in the ternary complex. Amino acid residues located at the TCR-MHC interface (distance cutoff <4 Å) are displayed as sticks, interacting H2-D^b^ residues are shown as semi-transparent spheres, and other residues are presented as wires. Hydrogen bonds are depicted as dotted lines. **e**, Interface between Hsf2 p.K72N_68–72_. Atoms presented as Van-der-Waals spheres, vdw) and TCR-47BE7 the protein surface is colored to discriminate between TCR chains.

**Fig. 4 |:**
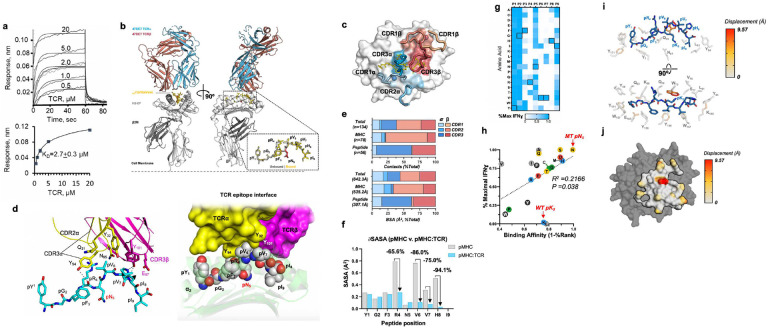
Structure of the pMHC-TCR ternary complex. **a**, Kinetics of TCR-47BE7 binding to immobilised H2-D^b^/Hsf2 p.K72N as determined by biolayer interferometry. Disassociation constant (K_D_) determined by curve fitting. **b**, Crystal structure of TCR-47BE7/H2-D^b^/Hsf2 p.K72N_68–76_. (Inset) Superposition of Hsf2 p.K72N_68–76_ peptide from bound (binary), and bound (ternary) crystal structures. **c**, En face view of H2-D^b^/Hsf2 p.K72N_68–76_ with superimposed TCR-47BE7 CDR loops. Surface is colored according to observed CDR1α-CDR3α (blue), CDR1β-CDR3β (red), or no TCR contact (white). **d**, (Left) The interface between bound neoepitope and TCR-47BE7 in the pMHC-TCR structure in cartoon model. The polar bonds are depicted as dotted lines. Only the TCR residues that are in direct contact (<4Å distance) with epitope atoms are shown as stick models. (Right) Interface between Hsf2 p.K72N_68–76_ (atoms presented as Vander-Waals spheres, vdw) and TCR-47BE7 (the protein surface was colored according to the TCR chains). **e**, Percent of total TCR contacts, TCR-MHC contacts and TCR-peptide contacts (top) and percent of total buried surface area (BSA), MHC BSA and peptide BSA (bottom), derived from each CDR loop shown in **c. f**, Residue-specific solvent-accessible surface area (SASA) in unbound/binary (gray) and bound/ternary (blue) structures. **g**, CD8^+^TCR-47BE7^+^ T cells were incubated with 1 μM YGFRNVVHI peptide variants from the positional scanning library, along with aCD28, for 6 hours and IFNγ production measured by intracellular flow cytometry staining. Cell color indicates mean of biologic replicates (n=2). Boxed squares indicate native amino acid at indicated position in mutant peptide sequence YGFRNVVHI. **h**, Dot plot of position 5 peptide variants from the positional scanning library plotted according to in silico MHC-I binding affinity and measured %IFγ^+^TCR-47BE7^+^CD8^+^ T cells by ICS (D). Colored according to side chain biochemical property. Polar uncharged (yellow), Positive charge/acidic (red), negative charge/basic (blue), hydrophobic (grey), aromatic (black), special (green). MT= mutant, WT= wild type. **i**, Stick representative docked structure of H2-D^b^/Hsf2_68–76_ with critical interacting H2-D^b^ residues shown. Atoms are colored according to mean root mean squared deviation (RMSD) from H2-D^b^/Hsf2 p.K72N_68–76_ crystal structure, scaled from 0Å (white) to 9.57Å (red). **j**, En face view of H2-D^b^/Hsf2_68–76_.TCR-47BE7 contact surface is colored according to mean root mean squared deviation (RMSD) from H2-D^b^/Hsf2 p.K72N_68–76_ crystal structure.

Having identified a plausible physical mechanism for enhanced binding of the neoAg Hsf2 p.K72N_68–76_ to H2-D^b^, we sought to understand the physical basis for TCR recognition. We hypothesised that TCR recognition was mediated by the structural interactions between the TCR and mutant peptide specific structural feature dependent on the pN5 anchor, the solvent-exposed p6-p8 ridge. TCR 47BE7 (V_α_7–1:J_α_21, ν_β_2Ή_β_2:_β_2–1) is derived from a vaccine-induced cytotoxic CD8^+^ T cell clone that recognises the H2-D^b^/Hsf2 p.K72N_68–76_ with sub-nanomolar functional avidity (EC_50_ 5.61pM, 95%CI 5.15–6.11pM). To understand the structural basis for 47BE7 binding, we synthesised recombinant soluble TCR as previously described^38^. Correct folding and preserved substrate recognition in solution were determined by measuring interaction kinetics between 47BE7 and H2-D^b^/Hsf2 p.K72N_68–76_ by biolayer interferometry (BLI). 47BE7 bound immobilised H2-D^b^/Hsf2p.K72N_68–76_ with affinity typical for non-self-reactive TCR (K_D_ 2.7±0.3 μM) (Fig. 4a)^39^. Complex *on* and *off*-rates were too fast to be determined with precision. To identify the physical basis for TCR binding, we crystallised and solved the 47BE7/H2-D^b^/Hsf2 p.K72N_68–76_ ternary structure to 2.6Å (Fig. 4b). The crystal structure exhibited defined electron density at the TCR:pMHC interface, allowing unequivocal placement of all critical amino acid side chains at the complex interface. TCR 47BE7 exhibited conventional oblique docking geometry (13.13° incident-angle, 57.49° crossing-angles). The TCR centroid was biased towards the peptide C-terminus, overlying the solvent-exposed and structurally rigid p6-p8 segment identified in the pMHC binary structure and hypothesised to contribute to TCR binding (Fig. 4c). The total interface BSA was 842.3 Å^2^ (364.9 Å^2^ TCRα, 477.4 Å^2^ TCRβ); of which 63.54% and 36.46% was made with H2-D^b^ and peptide, respectively. Interfacial contacts between 47BE7 and H2-D^b^/Hsf2 p.K72N_68–76_ were mediated by complementarity determining region (CDR) loops CDR1α, CDR2α, CDR3α, CDR2β, and CDR3β, with little contribution from CDR1β. (Fig. 4d,e). The bound and unbound structure of H2-D^b^/Hsf2 p.K72N_68–76_ was relatively preserved (0.6Å Cα RMSD), arguing against gross structural re-organization of the peptide-MHC on TCR complexation (Extended Data Fig. 3a). The buried residue H2-D^b^ Y_159_ projected into the *Epocket* in the unbound structure but was observed to rotate 83.54° towards the *D pocket* in the bound structure, and the space previously occupied by Y_159_ is instead filled with glycerol (Extended Data Fig. 3b). The presence of the glycerol contaminant in this position did not significantly alter other amino acid side chains, preserved the expected p6-p8 arched peptide confirmation, and had no appreciable impact on the TCR interface.

Binding of the TCR to H2-D^b^ was mediated by the solvent-exposed residues H2-D^b^ Q_72_, R_75_, R_80_ (H2-D^b^ α_1_ helix), as well as G_18_-E_19_ (H2-D^b^ loop A) which undergo re-organization on complexation. (Extended Data Fig. 3c). The H2-D^b^ loop A translates 4.8A, and concomitantly the side-chain of E_18_ rotates 161.14° vis-à-vis the backbone Cα towards while E_19_ rotates 104° away from the H2-D^b^ α_1_ helix. This places E_18_ within the hydrogen bond distance of R_75_ and R_79_, which positions R_75_ to form hydrogen bonds with the hydroxyl and carboxyl groups of CDR2β S_51_ and the Y_52_ carboxyl (Extended Data Fig. 3d). Additional interfacial TCR-MHC polar interactions include hydrogen bonds between CDR2β M_56_-Q_72_ and CDR3β Q_97_ and N_80_, as well as additional salt bridges between CDR3β E_97_ and K_146_ (H2-D^b^ α2 helix). Polar interactions between TCRα and H2-D^b^ were limited to a single hydrogen bond between CDR1α Y_32_-S_150_ (H2-D^b^ α2 helix) (Extended Data Fig. 3d).

As expected, binding of the TCR to the Hsf2 p.K72N_68–76_ peptide was mediated by the C-terminal epitope comprised of pV_6_-pH_8_, which formed a rigid solvent exposed arch. In agreement, a comparison of solvent-exposed surface area (SASA) by peptide residue of the bound and unbound pMHC complex show significant SASA reduction for pR4 and pV_6_-pH_8_, which comprise the peptide contribution to the core epitope buried by TCR 47BE7 (Fig. 4f). For the remaining peptide residues—including the mutated residue pN_5_—SASA does not change significantly on complexation, suggesting these side-chains do not contribute to ternary complex formation. Additionally, the observed polar interactions were limited to the CDR3α Y_94_ hydroxy and the pR4 backbone carboxyl; the CDR3β Y_101_ hydroxyl and the pH_8_ side chain imidazole and backbone amide; as well as a salt bridge between CDR3β E_97_ and pH_8_. Other electrostatic interactions were observed in the form of water bridging between CDR2β D_57_ as well as CDR3β N_95_ and pR_4_. Finally, non-polar van der Waals (VdW) contacts form between CDR1α Q_31_ and Y_32_ and pV_6_ and, as well as CDR3α Y_32_, CDR3β Y_101_ and pVal_7_. Collectively, these data suggest that the TCR 47BE7 epitope is biased towards the peptide C-terminus, in a region of pre-existing structural rigidity within H2-D^b^/Hsf2 p.K72N_68–76_ binary complex. The epitope surface chemistry is defined by sparse peripheral polar contacts with solvent-exposed amino acids pR_4_ and pH_8_. Centrally, there is a densely packed hydrophobic patch stabilised by multiple VdW interactions with the peptide arch formed by sequential pV_6_-pV_7_ residues. (Extended Data Fig. 3e). Surprisingly, while the mutant pAsn_5_ residue lies within the core epitope, it makes no significant side-chain contacts with TCR-47BE7, suggesting that it has a minimal direct impact on TCR binding. Instead, the pN5 anchor *indirectly* stabilises the critical components of the core epitope required for TCR binding.

## Structural Basis for Neoantigen-specific TCR Selectivity

Selectivity for the mutant peptide is an important characteristic of neoantigen-reactive TCRs that is hypothesised to provide an increased therapeutic window relative to TCR responsive to non-mutated tumour antigens. In cell-based cytokine production assays, we found TCR 47BE7 to be approximately 1.55×10^6^ fold more sensitive to the mutant peptide (EC_50_, 5.6pM, 95%CI, 5.2–6.1pM), relative to the WT peptide (EC_50_, 8.7μM 95%CI, NR) (Fig. 2d). This difference was several orders of magnitude larger than the 175-fold difference in binding affinity observed in RMA-S MHC-I stabilization assays (Fig. 3a). Moreover, in response to saturating peptide concentrations, we observed significantly greater cytokine production on a per-cell basis when exposed to MT versus the WT peptide, suggesting that the WT peptide functions as a partial agonist only. Notably, TCR 47BE7 binds via an epitope comprised of the solvent-exposed peptide residues [X]- [X]- [X]-pR_4_-[X]-pV_6_-pV_7_-pH_8_-[X], which are conserved between Hsf2 p.K72N_68–76_ (YGFR**N**VVHI) and Hsf2_68–76_ (YGFRKVVHI). The non-conserved MT residue pN_5_ side-chain provides stabilization to the pMHC but is not essential for peptide-presentation and does not directly contribute to TCR bond formation (Fig. 4d). This suggests that secondary structural factors indirectly related to the p.K72N amino acid substitution, such as stabilization of the pV_6_-pH_8_ arch, contribute to antigen discrimination by TCR-47BE7.

**Extended Data Fig. 4 |:**
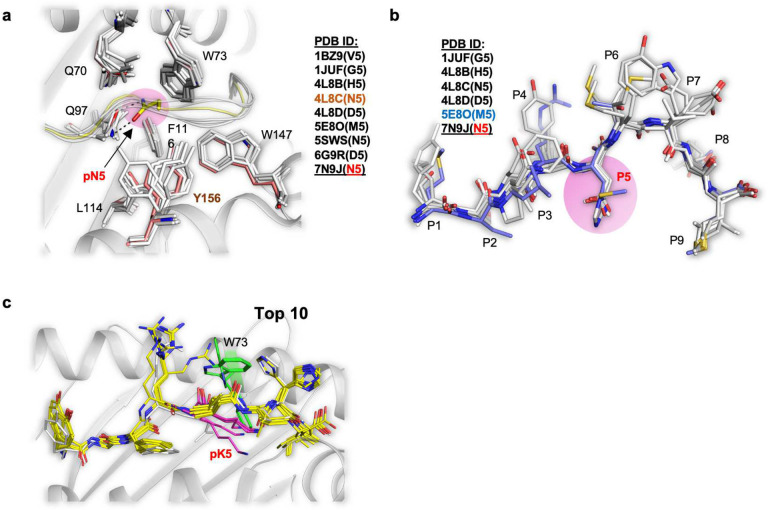
Additional structural diagrams depicting the pMHC-TCR ternary complex. **a**, Superimposition between atomic coordinates of the H2-D^b^ crystal structures from the PDB in complex with various epitopes, cartoon and sticks, Hsf2 p.K72N_68–72_ carbon atoms are shown in yellow. The PDB files are listed in the figure; amino acid residue in peptide positions shown in parentheses. 7N9J is the structure of H2-D^b^ in complex with Hsf2 p.K72N_68–72_ epitope described here. **b**, Superimposition between atomic coordinates of the various peptides complexed with H2-D^b^, stick representation. The PDB files are listed in the figure, in parentheses, the amino acid residue at P5 is shown. **c**, Superimposition of the top 10 solutions for each docking scenario, cartoon representation. Peptides are shown as sticks. Peptide carbon atoms (except for pLys_5_) are yellow, the H2-D^b^ side chain carbon atoms are colored in orange, nitrogen atoms are blue, oxygen atoms are red. H2-D^b^ Trp_73_ is colored green.

To further characterise the biochemical basis for antigen discrimination we created a positional scanning peptide library. Each position in Hsf2 p.K72N_68–76_ was replaced with the remaining 19 protein-coding amino acids then cytokine production by 47BE7-expressing tgTCR CD8^+^ T cells was assessed (Fig. 4g). Side-chain substitution of the non-core anchor residues pY_1_-pG_2_ and pI_9_ were generally tolerated. A limited set of conservative, primarily aromatic substitutions was tolerated at the non-core residue pF_3_. Therefore, these residues are non-essential for TCR binding. Conversely, side-chain substitutions within the core epitope pArg_4_-[X]-pVal_6_-pVal_7_-pHis_8_ abolished TCR recognition. Exceptions included biochemically conservative substitutions for the polar-basic residue pR_4_ (Q, T) and non-polar residues pV_6_ (I, Y) and pV_7_ (L, M). Notably, 47BE7 tolerated multiple side-chain substitutions at position 5. This was surprising given known H2-D^b^-mediated constraints favouring the *secondary* anchor pN_5_. This observation could be explained by both the lack of significant interfacial contacts between 47BE7 and the side chain of pN_5_, as well as the possibility that additional P5 residues may allow stabilization of the core epitope. Consistent with this, analysis of available PDB H2-D^b^ structures demonstrates that alternative p5 residues, including glycine, alanine, aspartate, histidine and methionine, adopt conformations similar to that of asparagine (Extended Data Fig. 4a,b).

Hypothesizing that a stable peptide-MHC interaction is necessary for TCR binding, we performed *in silico* binding affinity analysis of all position 5 substituted peptides. We observed a weak direct correlation between predicted binding affinity to MHC-I and TCR-47BE7 reactivity. Notably, the WT Hsf2_68–66_ (YGFRKVVHI) was a significant outlier to this trend, suggesting differential peptide-MHC binding affinity alone was not sufficient to explain the selectivity of TCR-47BE7 (Fig. 4h).

To identify alternative structural explanations, we performed *in silico* docking studies of Hsf2_68–76_ using the H2-D^b^/Hsf2 p.K72N binary structure as a template as previously described^6^. Initial models suggested that the H2-D^b^ *C pocket* is unable to accommodate the large pK_5_ without steric clashes with H2-D^b^ residues lining the binding pocket. Thus, binding of WT Hsf2_68–76_ requires reorganization of the H2-D^b^ binding pocket relieving steric clashes. (Extended Data Fig. 4c). In the most stable docked H2-D^b^/Hsf2_68–76_ models the pLys5 side chain assumed an extended conformation with the basic side chain buried projected towards the *Fpocket*. In this configuration the ensemble of docked H2-D^b^/Hsf2_68–76_ was compatible with stable pMHC structures (Rosetta energy unites [REU], −491.74±2.51SD), and exhibited largely conserved Cα structure (RMSD 0.724 ±0.20 SD) with H2-D^b^/ Hsf2 p.K72N_68–76_. Importantly, while buried, pK_5_ does not permit formation of the characteristic hydrogen bond network with H2-D^b^ N_97_. Additionally, the extended pK5 is sterically incompatible with H2-D^b^ W_73_ (α1 helix). As a result, W_73_ which is buried in the reference structure (SASA 5.695 Å^2^), rotates 180° (9.57Å) to become solvent-exposed (53.137 Å^2^, SASA) (Fig. 4i). This rotation disrupts the conserved H2-D^b^ W_73_-W_147_-Y_156_ bridge and changes the surface topology and hydrophobicity in this region of the core TCR epitope (Fig 4j). Specifically, in the TCR-47BE7/H2-D^b^/Hsf2 p.K72N_68–76_ ternary crystal structure the affected area is a cavity bounded by the pR_4_ and pH_8_, H2-D^b^ E_72_ and R_75_ which collectively coordinate four water molecules. We previously observed these water molecules to form water bridges with TCR-47BE7 CDR303α N_95_. Rotation of the H2-D^b^ Trp_73_ hydrophobic side-chain into this position likely expels these waters and abrogates electrostatic interactions between CDRα N_95_ and H2-D^b^ E_72_. Collectively, our modeling suggests that TCR-47BE7 specificity towards H2-D^b^/Hsf2 p.K72N is not solely attributable to robust peptide-MHC stability, but also to changes in surface topology and hydrophobicity at the TCR-peptide/MHC binding interface.

## Discussion

There is significant interest in the study of neoantigens and corresponding reactive TCR due to published association with clinical outcomes in patients treated with tumour immunotherapy. However, clinical translation of these findings is limited by the lack of relevant pre-clinical models for testing fundamental assumptions of neoantigen biology. The experiments we present were designed to provide a preclinical model for studying neoAg-reactive TCR structure-activity relationships.

We first identified and performed a basic characterization of several TCR-antigen combinations from the widely-utilised B16F10 melanoma model and present these to the community for further study. We then completed in-depth biochemical and structural studies of the prototype anchor-residue modified neoantigen H2-D^b^/Hsf2 p.K72N and the corresponding monoclonal TCR 47BE7. We selected H2-D^b^/Hsf2 p.K72N_68–76_ and 47BE7 for characterization due to demonstrable *in vitro* and *in vivo* activity in a challenging tumour model. Furthermore, 47BE7 exhibited high functional avidity and limited cross-reactivity in our *in vitro* studies, which we hypothesised could provide insight into mechanisms underlying binding and cross-reactivity of neoAg-reactive TCRs.

We found that the lysine to asparagine substitution at the position five anchor residue results in a 175-fold improvement in the surface presentation of H2-D^b^/Hsf2 p.K72N_68–76_ relative to the wild-type Hsf2_68–76_. The crystal structure of H2-D^b^/Hsf2 p.K72N demonstrates that the mutated pAsn_5_ residue is directly responsible for this effect, due to stabilizing polar interactions between pN_5_ and H2-D^b^ N_97_. As an additional direct consequence of the anchor residue mutation, the carboxy-terminal segment of the neoAg peptide distal to pAsn5 forms a rigid solvent-exposed hydrophobic arch which is essential for binding to TCR 47BE7. The structural stability of neoAg pMHC has been repeatedly associated with immunogenicity^29,30^.Our data suggest the plausibility of this association being driven by increased pMHC surface abundance secondary to slow peptide disassociation kinetics. We expand on these findings by showing that pMHC stability measures may indirectly capture fine structural features associated with immunogenicity, including rigid structural elements necessary for TCR recognition. We propose that when these unique pMHC structural features are generated by somatic amino acid substitutions at anchor positions, particularly when presenting hydrophobic features, they may explain the immunogenicity of class II neoAg. This model may permit positive selection and peripheral maintenance of neoAg-reactive TCRs, while limiting the effects of negative selection. Notably, H2-D^b^/Hsf2 p.K72N exhibits features strikingly similar to a H2-D^b^-restricted pN_5_ influenza peptide/MHC complex previously characterised, in which a hydrophobic ridge in H2-D^b^ enables solvent accessibility of residues at peptide positions 4, 6 and 7. These parallels suggest that neoantigen Hsf2 p.K72N behaves similarly to a viral-derived peptide^34^, providing structural evidence for the longstanding hypothesis that neoantigens are recognised by T cells as foreign peptides.

Recently, several groups have published structural studies of human neo-reactive TCR^22,24,40^. The structural data we present supports and expands on these earlier findings in several noteworthy ways. First, we observed high-level commonalities between TCR 47BE7/Hsf2 p.K72N and TCR9a/TCR10, as well as TCR4, which bind to the class II (anchor-residue modified) neoAg HLA-C*08:02/Kras p.G12D^22,41^. These TCRs employ a similar binding mode characterised by multiple intermolecular contacts distributed across the TCR:pMHC interface. Experimental modification of contacted residues within the core TCR epitope eliminates TCR reactivity, suggesting that the totality of the interface is necessary in both instances for TCR binding, similar to Hsf2 p.K72N. This binding mode contrasts notably with that employed by neoAg-reactive TCR that bind to class I (solvent-exposed residue modified) neoAg such as TCR12–6/TCR38–10, which bind to the neoAg HLA-A*02:01/TP53 p.KR175H^39^. In this latter circumstance the observed TCR contacts are biased towards the solvent-exposed mutant residue and avoid contacts with the remaining peptide surface. While we observe some bias towards the peptide C-terminus in the footprint of TCR 47BE7, the eccentricity is not as extreme as has been described for class I neoAg. These observations, while speculative, suggest that there may be stereotyped binding modes exhibited by neoAg-reactive TCR targeting class I and class II neoantigens, and by extension, predictable, albeit noisy, rules governing neoAg immunogenicity. Further elucidation of the rules governing these interactions may enable rapid clinical translation of safe and effective neoAg-reactive TCRs.

## Methods

### Cell Culture

B16F10 (#: CRL-6475) and RMA-S (#: TIB-39) cells were purchased from American Type Tissue Culture (ATCC). Platinum-E (#: RV-101) cells used for retroviral packaging were purchased from Cell Biolabs. Upon arrival, cells lines were tested regularly for mycoplasma (Lonza, cat#: LT07–318), and rodent pathogens by IMPACT (IDEXX), and reference cell banks were generated. All cell lines were maintained Dulbecco’s Modified Eagle’s Medium (DMEM) with GlutaMAX^™^, HEPES 20mM, penicillin-streptomycin and fetal bovine serum (FBS) 10%v/v at 37°C in a 5% CO_2_ humidified atmosphere.

### Whole Exome Sequencing

B16F10 (tumour) cells were expanded in culture to 75% confluence. Total splenocytes (germline) were isolated from a male C57BL/6 colony founder. Genomic DNA was isolated using DNeasy Blood & Tissue kit (Qiagen, cat#: 6950). Whole exome sequencing (WES) libraries were prepared using SureSelectXT Mouse All Exon kits (Agilent, cat# G7550A). Paired-end, 100bp sequencing was performed using HiSeq2500 reagent kit v3 (Illumina, CA) targeted sequencing depths of 300x and 150x for tumour and germline samples, respectively. Sequencing reads were mapped to GRCm38.p6/mm10 using BWA-MEM(37). Duplicate read marking and base quality score recalibration was performed using GATK/Picard(42). Somatic variant calling was performed for target regions using MuTect and Strelka with default filters(43).

### Isolation of Tumour mRNA and RNA Sequencing

B16F10 cell tumour cells (1×10^6^) were inoculated into the dermis of subject animals. Seven days post-inoculation the tumours were resected, and total RNA was isolated using RNeasy kits (Qiagen, cat#: 74104). Messenger RNA sequencing library generation was performed using Ribo-zero magnetic gold and TruSeq RNA Sample preparation kits (Illumina, CA). Paired-end, 100bp, sequencing was performed using a HiSeq 2500 reagent v3 kit, with a targeted sequencing depth of 1×10^8^ reads/library. Sequencing reads were mapped to GRCm38.p6/mm10 using HiSa.

### Identification of Mutation-derived Tumour Neoantigens (neoAg)

Mutation-derived tumour neoantigens were identified as previously described(25). Briefly, somatic variants are identified by WES. Variant expression is quantified by local assembly and allele-specific quantification of mutated and reference transcripts. Variant transcripts are translated *in silico*. The peptide-MHC-I binding prediction tool NetMHCpan (v.4.1) was then used to identify candidate neoAg for further study.

### Peptide synthesis

Experimental peptides were individually custom synthesised via the solid-phase method by GenScript (Piscataway, NJ), with standard removal of trifluoracetic acid and replacement with hydrochloride, purified to > 98% by HPLC, and lyophilised for storage. Peptides were reconstituted in DMSO at 10 μM and frozen at −80°C until use.

### Immunization

Peptide-based vaccines comprising peptide antigen and TLR7/8a adjuvant co-delivered in self-assembling particles (referred to as “SNAPvax^™^”) were produced as previously described (27–28). Briefly, peptide antigens, synthesised as described, were linked to a hydrophobic oligomeric peptide linked to imidazoquinoline-based TLR7/8a (Vaccitech North America, USA) using a click chemistry reaction. Vaccines were reconstituted in sterile phosphate-buffered saline to a final concentration of 40 μM, and 50 μL was injected subcutaneously to bilateral footpads.

### Mice

C57BL/6J (C57BL/6) and C57BL/6-Tg(Nr4a1-EGFP/cre) 820Khog/J (Nr4a1-eGFP) were purchased from Jackson Laboratory (Bar Harbor, ME). Mice were housed in a specific pathogen-free (SPF) containment facility located at the Icahn School of Medicine at Mount Sinai. Procedures and monitoring protocols were approved by the Icahn School of Medicine at Mount Sinai Institutional Animal Care and Use Committee (IACUC) protocol: 15–2171, approval: IACUC-2016–0028.

8–12 week-old animals balanced with respect to age and sex were used for all immunization, adoptive cell transfer, and tumour allograft experiments. Mice 8–12 weeks old, balanced for age and sex, were used for all immunization, adoptive cell transfer, and tumour allograft experiments. Subjects were evaluated every 48 hours for the full duration of all experiments. Euthanasia was performed by carbon dioxide asphyxiation followed by cervical dislocation.

Peripheral blood was obtained by submandibular vein puncture. Approximately, 250 μL of was collected into sterile heparinised tubes. Red blood cells were removed with ammonium-chloride-potassium (ACK) osmotic lysis solution; 2:1 v/v for 5min, followed by centrifugation at 500 × g, 5min. The resulting peripheral blood mononuclear cells (PBMC) were washed twice with MACS buffer (PBS, BSA 2%v/v, EDTA 2 mM), and then stored in MACS buffer at 4°C until use.

Total splenocytes were obtained by splenectomy. Tissue samples were macerated over 70 μm pore-size nylon filters. Red blood cells were removed by treating the samples with ACK lysis solution; 2:1 v/v for 5min, followed by centrifugation at 500 × g, 5min. Total splenocytes were washed twice with MACS buffer (PBS, BSA 2%v/v, EDTA 2 mM) and maintained at 4°C until use.

### Tumour Inoculation

Orthotopic injections of B16F10 melanoma cells were performed as described previously(44, 45). Briefly, subjects were sedated and paralyzed by administration of 1:2:7 %v/v xylazine: Ketamine: deionised water, 100 μL, intraperitoneal (IP) injection, once. Mechanical shears were used to remove hair from a 2 cm^2^ body surface area overlying the posterior hindlimb. Sterile 70% v/v Ethanol: deionised water swabs were used to remove disinfect the skin surface. Tumour cell suspensions containing 100,000 cells in 100 μL were loaded into syringes with permanent 28G needles. Needles were inserted into the skin to the level of the dermis and the full volume was injected. Subjects were returned to the enclosure and monitored for complete recovery from anesthesia. Humane endpoints for subject withdrawal were as follows: tumour diameter ≥15 mm, ulceration or body condition score <3 (ordinal scale).

### RMA-S MHC-I Thermostability Assay

H-2 stabilization experiments were performed as previously described(46). Briefly, RMA-S cells were placed in culture at 25°C 5% CO_2_ for 18 hours, then incubated peptides at the stated concentration for 30 min. at 30°C 5% CO_2_, then incubated for 3 hours at 37°C 5% CO_2_. Cells were then washed twice with PBS, stained with fluorophore-conjugated monoclonal antibodies specific to H2-K^b^ (Clone: AF6–885, Biolegend) or H-2D^b^ (Clone: KH95, Biolegend) [0.5μg/mL], 4°C, 30min, washed twice with PBS, fixed with PFA 1% w/v. Data acquired on BD LSRFortessa.

### MHC Tetramer Staining

MHC tetramer staining was performed as previously described(47). Briefly, MHC tetramer reagents were non-covalently linked to streptavidin-PE (Invitrogen, cat#: S866) and/or streptavidin-APC (Invitrogen, cat#: S868) 1:1 mol:mol. Single-cell suspension of total PBMC/splenocytes or isolated CD8^+^ T cells were suspended in PBS (FCS 2% w/v, EDTA 2mM) supplemented with dasatinib (SelleckChem, cat#: S1021) 50nM, then incubated 30min, 20°C. MHC-tetramer (100nM), and anti-mouse CD8a (2.5× 10^−4^)g/L, Clone: CT-CD8a) was added then incubated, 60min, 4°C. The cells were then washed with PBS (FCS 2%, EDTA 2mM) twice, then suspended in PBS (Paraformaldehyde, 1% w/v) and stored at 4°C until use.

### Antigen-specific T Cell Clones and TCR Sequencing

CpG-C/ODN-2395 (_5_T*C*G*T*C*G*T*T*T*T*C*G*G*C*G*C*G*C*G*C*C*G) was produced by IDT. Subject animals were treated with vaccines as described above. Six days post-immunization a single-cell suspension of MutuDC cells 1×10^5^ cells, 100 μL , (1×10^9^ cells/L) was added to round-bottom 96-well microtiter plate wells, then incubated for 37°C, 24h. IMDM FCS 8% v/v, supplemented with peptide 2×10^−6^ M, CpG-C/ODN-2395 and recombinant murine IFN (PeproTech, Cat#: 315–05) 1×10^5^ U/L were added, then incubated at 37°C, 3hr. MutuDC cells were irradiated to a final dose of 50 Gy, then culture media was changed to RPMI FCS 10%v/v, 1×10^−3^ L, 1×10^6^ cells (1×10^9^ cells/L) supplemented with 2-mercaptoethanol (5×10^−5^ M), recombinant human IL-2 (PeproTech, Cat#: 200–02) 2×10^5^ IU/L, recombinant murine IL-7 (PeproTech, Cat#: 217–17) 1× 10^−5^ g/L and recombinant murine IL-15 1×10^−5^ g/L (PeproTech, Cat#: 210–015).

Seven days post-immunization CD8^+^ T cells were isolated from total splenocytes by bead-based affinity chromatography (Miltenyi, Cat#: 130–104-075). MHC tetramer staining was performed as described above. Single tetramer^+^ T cells were sorted onto peptide-pulsed irradiated MutuDC feeder-layers using a FACS Aria III (BD), then incubated at 37°C, for 5–7d. Single-cell cultures were periodically visually assessed by bright-field microscopy for viability and cell expansion. Wells demonstrating secondary expansion were split, as necessary to maintain cell concentration (1×10^6^ cells/well). MHC tetramer staining was performed on expanded clonal T cell lines to confirm antigen-specificity.

Total RNA was isolated using RNeasy Micro Kit (Qiagen, cat#: 74034) according to manufacturer specifications. Paired 5’ RACE TCR sequencing libraries were generated using SMARTer Mouse TCR a/b Profiling Kit (Takara, cat#: 634403), according to manufacturer specifications. Library insert size was determined by Bioanalyzer DNA 1000 Kit (Agilent, cat#: 5067–1504). Library sequencing was performed using MiSeq Sequencer (Illumina, CA), 300bp, paired-end, with targeted sequencing depth of 2×10^7^ reads/library. Demultiplexed FASTQ files were assembled into full-length TCR cDNA sequences using MiXCR(48).

### Intracellular Cytokine Staining

Intracellular cytokine staining was performed as previously described (49). Surface mobilization of CD107a was measured as previously described (50). Briefly, isolated lymphocytes were cultured in RPMI (FCS 10%v/v) supplemented with anti-CD107a (Clone: Lamp-1, BioLegend), anti-CD107b (Clone: Mac-3, Biolegend), anti-CD28 (Clone 37.51; BioXCell); peptide, 2μM; GolgiPlug (BD, cat#: BDB555029) 1×10^−3^g/L; GolgiStop, cat#: 554724) 1×10^−6^M; for 6h, 37°C. The cells were then washed, stained with LIVE/DEAD fixable viability dye (ThermoFisher, cat#:), 30m, 20°C. Then stained with anti-CD3 (17A2, BioLegend) anti-CD8 (Clone 53–6.7, BioLegend), anti-CD4 (RM4–5, Biolegend), 30min, 4°C. The cells were washed, and suspended in Fix/Perm solution (BD, Cat#: 554715) then incubated for, 30min, 4°C. The cells were washed twice in Perm/Wash solution (BD, Cat#: 554715), suspended in Perm/Wash solution containing anti-IFNγ (Clone: XMG1.2, Biolegend), anti-IL-2 (Clone:JES6–5H4, Biolegend), anti-TNFα (Clone: MP6-XT22,Biolegend) then incubated, 30min, 20°C. Cells were then washed twice and suspended in PBS (PFA 1%w/v) and stored at 4°C until use.

### B16F10:T cell Co-culture

B16F10 cell tumour cell suspension consisting of 1×10^5^ cells (1×10^9^ cells/L) 1×10^−5^L DMEM FCS 10%v/v was added to 96-well flat bottom plates, then incubated 37°C, 8h. 1×10^−5^L DMEM FCS 10%v/v supplemented with recombinant murine rmIFNγ 5×10^4^U/L was added, then incubated 37°C, 12–16h. Media was replaced with single-cell suspension of CD8^+^ T cells (1:1 ratio T cell:B16F10) in RPMI FCS 10%v/v, supplemented with anti-CD28 (Clone 37.51; BioXCell), then incubated 6h at 37°C. T cells were removed with gentle pipetting, then washed twice with PBS FCS 2%v/v, EDTA 2mM before use.

### Retrovirus Plasmids for T cell Transduction

pEF-ENTR A (696–6), pLenti X1 Zeo DEST (668–1), pLenti X1 Puro DEST (694–6), and pLenti X1 Zeo DEST (668–1) were gift from Eric Campeau & Paul Kaufman (Addgene# 17427, 17299 and 17297). pMSCV-IRES-GFP II (pMIG II) was a gift from Dario Vignali (Addgene# 52107). MSCV-IRES-Thy1.1 DEST was a gift from Anjana Rao (Addgene# 17442). pCL-Eco was a gift from Inder Verma (Addgene# 12371).

pMSCV(v5) γ-retrovirus transfer plasmids were constructed based on pMIG II, with the following modifications. pMIG II was linearised with EcoRI-HF (NEB, Cat#: R3101S), and AgeI-HF (NEB, Cat#: R3552S). The Woodchuck Post-transcriptional Regulatory Element (WPRE) from pLenti X1 Puro DEST (694–6), as well as the CD90.1/Thy1.1 from MSCV-IRES-Thy1.1 DEST were amplified by PCR. The complete cDNA for TCRα, a furin (R/Arg-A/Ala-K/Lys-R/Arg) cleavage target and the Thosea asigna virus 2A (T2A), followed by the complete cDNA for TCRβ, a furin cleavage target and porcine teschovirus-1 2A (P2A) were synthesised (Genscript, NJ)(51). Point mutations in TRAC [p.T48C] and TRBC1/2 [p.S57C] were introduced to promote receptor pairing(52, 53). Segments were assembled in series into the linearised pMIG II backbone by flanking homology (NEBuilder HiFi DNA Assembly, NEB). Sanger sequencing (Genscript, NJ) was used to verify the correct sequence, order, and orientation of all constructed plasmids.

### Lentivirus Plasmids for Creation of Tumour Antigen-overexpressing Tumour Lines

Antigen-overexpressing tumour lines were engineered to normalise antigen levels; notably, Hsf2 is expressed at lower levels than non-mutated tumour antigens studied (54). pENTR Gateway DONOR plasmids were constructed as follows. pEF-ENTR-A (696–6) was linearised with BamHI (NEB, Cat#: R3136S))and EcoRI-HF (NeB, Cat#: R3101S). The cDNA for mTagBFP2; G/Gly-S/Ser-G/Gly spacer; T2A; followed by the cDNA corresponding to the 25 amino acid segment surrounding indicated neoantigen peptides and a c-terminal flag (-DYKDDDDK) tag were synthesised (Genscript, NJ). Segments were assembled in series into the linearised pENTR-A backbone using the flanking homology method (NEBuilder HiFi DNA Assembly, NEB). pLenti transfer vectors were generated using LR Clonase II (ThermoFisher, Cat#: 11791020). Sanger sequencing (Genscript, NJ) was used to verify the correct sequence, order, and orientation of all constructed plasmids.

### Viral Vector Production

Ecotropic γ-retrovirus (γRV) particles were produced by transient co-transfection of HEK293T Platinum-Eco (Platinum-E) cells as previously described (55). Briefly, Platinum-E cells were seeded in 6-well microtiter plate in 1.5×10^−3^L DMEM FCS 10%v/v, 1.2×10^6^ cells (1.27×10^5^ cells/cm^2^) then incubated at 37°C, 36h. Culture media was replaced with DMEM FCS 10%v/v omitting Penicillin-Streptomycin then incubated at 37°C, 60min. Transfection particles were prepared by mixing MSCV-based γRV transfer vector (pMSCV), and packaging vector (pCL-Eco) 2:1 mol/mol ratio with FuGENE 6 lipid transfection reagent (Lonza, Cat#: E2691) according to manufacturer specifications. Transfection particles were added dropwise to Platinum-E cells, then incubated at 37°C, 12h. Culture media replaced with DMEM (FCS 10%v/v, HEPES 20mM, GlutaMAX), then incubated at 37°C, 36Hr. Viral supernatant was collected at 48h and 72hr post-transfection and centrifuged at 1000 × g, 5min before use.

### Retroviral Transduction

γRV transduction of CD8^+^ T cells was performed as previously described(56). Briefly, 250 μL PBS containing Retronectin/rFN-CH) (Takara, Cat#: T100B) 2e-2g/L, was added to non-treated 24-well tissue culture dish, then incubated at 4°C, 12h. Retronectin solution was removed, and 500 μL PBS containing BSA 2%w/v was added, then incubated 20°C, 30min. BSA solution was removed, and 1 mL viral supernatant was added. The plate was sealed, then centrifuged 2000xg, 2h. CD8^+^ T cells, activated for 24h were centrifuged 500xg, 5m then suspended RPMI FCS 10%v/v, 1 mL, 1×10^6^ cells (1×10^9^ cells/L) supplemented with 2-mercaptoethanol (5×10^−5^M), rhIL-2 2×10^5^ IU/L. Viral supernatant was removed and the cell suspension was added, centrifuged 2000xg, 2h, then incubated at 37°C, 24h. Transduction was performed twice, 24h and 48h post-activation.

### Lentivirus Transduction

B16F10 cells were cultured in DMEM FCS 10%v/v to 75% confluence. TyrpLE (ThermoFisher, cat#: 12605010) was added, then incubated at 37°C, 10min. Cells were centrifuged, 500xg, 5min then re-suspended in 1.5×10^−3^ L DMEM FCS 10%v/v, 1×10^9^ cells/L and transferred to a 6-well microtiter dish. Lentivirus particles were added to 1.5×10^−5^ L DMEM FCS 10%v/v containing polybrene (EMDMillipore, cat#: TR-1003-G), 1×10^−1^ g/L. Lentivirus suspension was added to B16F10 cells, then incubated at 37°C, 24hr. Lentivirus supernatant was removed, and replaced with DMEM FCS 10%v/v, then incubated at 37°C, 24h. Lentivirus transduction was determined 48hr post-transduction by flow cytometry. Uniform populations of B16F10 mTagGFP2-minigene cells were isolated by fluorescence-activated cell sorting using a FACSAria III (BD Biosciences). Reference cell banks were generated on sort completion.

### CRISPR:spCas9 RNP Electroporation

CRISPR:Cas9 Ribonucleoprotein transduction of naive CD8^+^ T cells was performed as previously described(57), here to knockdown TCRα and TCRβ. CD8^+^ T cells were isolated from preparations of total splenocytes by negative selection using magnetic isolation beads (Miltenyi, Cat#: 130–104-075), according to manufacturer specifications. Cells were suspended 1×10^9^ cells/L in RPMI (‘RPMI FCS 10%v/v’, HEPES 20mM, GlutaMAX, Pyruvate, Non-essential amino acids, Penicillin-Streptomycin), supplemented with 2-mercaptoethanol (Gibco, cat#: 31350–010) 5× 10^−5^ M, recombinant murine IL-7 (‘rmIL7’, PeproTech, Cat#: 217–17) 1× 10^−5^ g/L, then incubated at 37°C, 12Hr.

Antibody-coated plates were prepared as follows. 2.5× 10^−4^ L PBS solution containing monoclonal antibodies specific containing anti-CD3 (BioXCell Cat#: BE0001–1) 1×10^−4^ g/L, and anti-CD28 (BioXCell Cat#: BE0001–1) 5× 10^−5^ g/L was added to each well of a 24-well tissue culture dish, then incubated at 4°C, 12Hr.

CRISPR:Cas9 Ribonucleoproteins (RNPs) were produced as follows. Synthetic CRISPR RNA (crRNA) and transactivating RNA (tracrRNA) were synthesised (IDT). Duplex crRNA:tracrRNA was produced according to the manufacturer’s specification, aliquoted, and stored at −80°C. RNP were produced by combining duplex RNA and TrueCut Cas9 Protein v2 (ThermoFisher, Cat#: A36498), 1.5× 10^−12^mol:5×10^−12^mol, then incubating at 20°C, 10min.

CD8^+^ T cells were washed twice with PBS then suspended in P4 Nucleofector solution (Lonza, Cat#: V4XP-4032) 2×10^−5^L, 1×10^−5^ cells (5×10^11^ cells/L). Alt-R Cas9 Electroporation Enhancer 1×10^−6^ L, 1×10^−4^ M (4×10^−6^ M) was added, followed by CRISPR:Cas9 RNPs. The cells were then transferred to 4D-Nucleofector X Unit (Lonza, Cat#: V4XP-4032). RNP were delivered by electroporation using 4D-Nucleofector (Lonza, cat#: AAF-1002X), pulse code: DS-137. The cells were then carefully distributed into a 96-well round-bottom tissue culture dish, containing RPMI FCS 10%v/v 2×10^−4^ L, 2×10^6^ cells/well (1×10^10^ cells/L), then incubated 37°C, 2Hr.

CD8^+^ T cells were transferred to RPMI FCS 10%v/v, 5×10^−4^ L, 1× 10^6^ cells (2× 10^9^ cells/L) supplemented with 2-mercaptoethanol (5×10^−5^ M), recombinant human IL-2 (‘rhIL-2’, PeproTech, cat#: 200–02) 2×10^5^ IU/L and recombinant murine IL-12p70 (PeptoTech, cat#: 210-12) 1×10^−5^g/L, then plated in 24-well plates coated with CD3/CD28 and incubated at 37°C, 24hr.

crRNA targeting TRAC and TRBC1/2 were designed with the IDT CRISPR-Cas9 guide RNA server (IDT), using reference genomic sequence of the TRBC1 (GRCm38.p6 C57BL/6J, ch6:41537984–41538423), TRBC2 (GRCm38.p6 C57BL/6J, ch1441546489–41547115), and TRAC loci (GRCm38.p6 C57BL/6J, ch14: 54219921–54224806) were used as target sequence references. crRNA were selected if PAM and/or crRNA nt p1–5 crossed an exon boundary. crRNA validation was performed by flow cytometry 72h post electroporation. CD8^+^ T cells exposed to RNP targeting TRAC, TRBC1/2 or were stained with CD3e-BV421 (17A2, Biolegend, cat#: 100228) CD8-FITC (53–6.7, Biolegend, Cat#: 100706), and TCRβ-PE (H57–597, Biolegend, Cat#: 109222), and isotype controls. crRNA achieving >90% reduction in surface CD3e/TCRb were retained. RNP-treated cells were assessed following transduction with γRV TCR-47BE7 and stained with H2-D^b^/Hsf2(47) pMHC tetramer. crRNA achieving >90% transduction, with surface expression of tgTCR determined by pMHC tetramer staining were retained. crRNA TRAC 5’-TCTGGGTTCTGGATGTCTGT PAM: GGG, and crRNA TRBC1/2 GTCACATTTCTCAGATCCTC PAM: TGG.

### CRISPR:spCas9 RNP Lipofection and Knockdown Cell Isolation

B16F10 cells were cultured in DMEM FCS 10%v/v to 75% confluence. TyrpLE (ThermoFisher, cat#: 12605010) was added, then incubated at 37°C, 10min. Cells were centrifuged, 500xg, 5min then re-suspended in 1.5×10^−3^ L DMEM FCS 10%v/v, 6.67×10^7^ cells/L and transferred to a 6-well microtiter dish. CRISPR:Cas9 RNP were prepared as described above, with the following modifications. RNP transfection particles were prepared using Lipofectamine RNAiMax lipid transfection reagent (ThermoFisher, cat#: 13778100), according to the manufacturer’s specifications. Transfection particles were added to 1.5×10^−3^ L DMEM FCS 10%v/v, supplemented with polybrene (EMDMillipore, cat#: TR-1003-G), then added to 6-well microtiter dish wells containing B16F10 single-cell suspensions. The combined mixture was incubated on an orbital shaker, 50 rpm, 5min, 20°C; then moved to incubate at 37°C, 5%CO2, 48h. Treated B16F10 cells were then split into two wells, containing 1.5mL DMEM FCS 10%v/v and maintained in culture to 75% confluence. Knockout of crRNA target gene products was measured by flow cytometry. Briefly, 1×10^−5^ L DMEM FCS 10%v/v supplemented with recombinant murine IFNγ (‘rmIFγ’, PeproTech, Cat#: 315–05) 5×10^4^ U/L was added to cultures, then incubated 37°C, 12–16h. B16F10 cells were disassociated from the culture dish by adding cold PBS containing EDTA 5mM, incubation at 20°C 5min, then gentle pipetting. Single-cell B16F10 suspensions were washed twice with PBS BSA 2%v/v, then PBS containing anti-H2-K^b^ (Clone: AF6–88.5, Biolegend) and anti-H2-D^b^ (Clone: KH95, Biolegend) or appropriate isotype control antibodies were added then incubated 4°C, 30min. Data acquisition was performed on LSR Fortessa (BD Biosciences). Target gene product surface protein expression level was defined using mock RNP-treated (positive control), and isotype control antibody (negative control) treated samples. To generate B16F10 H2-K^b−/−^ and H2-D^b−/−^ cell lines RNP-treated B16F10 cells were sorted with FACSAria III (BDBiosciences), re-verified by flow cytometry before being cryopreserved.

### Expression, folding and purification of H2-D^b^/YGFRNVVHI

Soluble peptide-MHC monomers were synthesised as previously described(58). Briefly, the extracellular domain of H2-K^b^, H2-D^b^, as well as full-length human B2M (hB2M) were cloned into pET3A plasmids (provided by Ton Schumacher, Netherlands Cancer Institute-Antoni van Leeuwenhoek (NKI-AVL)). For MHC tetramer production H2-K^b^ and H2-D^b^ expression constructs included a c-terminal biotin acceptor peptide (BirA-tag). Proteins were expressed in *E. coli* BL21(DE3) PLysS as inclusions bodies (IBs). IBs were solubilised in tris 100μM pH 8.0, supplemented with urea 8M, DTT 100μM, and EDTA 10mM. Insoluble material was cleared by centrifugation 20,000x g, 20min. Denatured proteins were added in a H2-D^b^:hβ2M 1:2 mol:mol ratio to solution of tris 100mM pH 8.0, supplemented with L-arginine 400mM, L-glutathione 500μM, oxidised L-glutathione 50μM, and EDTA 2mM, PMSF 10mM, protease inhibitor cocktail (Roche, Cat#: 11873580001) and peptide 1.2mM. The mixture was stirred at 4°C for 72h, and precipitate cleared by centrifugation at 20000 × g, × 10 min, concentrated by centrifugal ultrafiltration using a 15kDa membrane (Millipore, cat#: UFC903024) and sequentially purified by size exclusion chromatography (Superdex 75, GE Healthcare), followed by anion exchange (HiTrap Q HP 5, GE Healthcare). MHC monomers were concentrated by centrifugal ultrafiltration using a 4kDa membrane (Millipore, cat#: UFC8030) and exchanged into HBS (HEPES 10mM, NaCl 150mM). Peptide-MHC protein complex identity was verified by polyacrylamide gel electrophoresis, then split into single-use aliquots before snap-freezing in liquid nitrogen, then stored at −80°C until use. For MHC tetramer production site-specific enzymatic biotinylation by BirA biotin ligase was performed before the anion-exchange chromatography previously described.

### Expression, folding and purification of TCR 47BE7

Soluble TCR was synthesised as previously described(37). Briefly, αβTCR extracellular domains were ordered as codon-optimised synthetic DNA fragments. A stabilizing disulfide bond was engineered by introducing point mutations into the TCR constant domains [TRAC p.T48C, TRBC1 p.S57C] and a C-terminal Gly-Ser 6x histidine tag was appended to TRBC1. DNA fragments were cloned into linearised pET28a plasmids by Gibson assembly, then transformed into chemically *E. coli* BL21(DE3). Transformed cells were grown in Luria broth (LB) for 4–6 hours at 37°C shaking at 200rpm, induced with IPTG 100mM, then incubated for 4 at 37°C shaking at 200 rpm. Cells were pelleted by centrifugation at 4000x *g*, resuspended in lysis buffer (Tris-HCl 50mM, pH 8.0, sucrose 25%w/v, Triton-X100 1%v/v, EDTA 1mM, lysozyme (Sigma-Aldrich, Cat#: L6876) and DNAse (Roche, Cat#: 10104159001). Protein IBs were sequentially washed (Tris-HCL 20μM, pH 8.0, NaCl 150mM, Triton-X100 0.5% v/v, EDTA 1mM, DTT 1mM) then (Tris-HCL 20μM, pH 8.0, NaCl 150mM, EDTA 2mM, DTT 1mM), then solubilised (Tris-HCL 100μM, pH 8.0, Gdm-HCl 6M, DTT 10mM, EDTA 10mM). Denatured protein was added dropwise to a final 1:1 molar ratio (TCRα:TCRβ) to a folding solution (Tris-HCl 100mM, pH 8.0, urea 5M, L-Arg 400mM, L-GSH 5mM, L-GSSG 50μM, EDTA 2mM, PMSF 10mM). The mixture was stirred at 4°C for 72h, then cleared of precipitate by centrifugation at 20,000 × g, x10min. Then dialyzed (Tris-HCl 10mM, pH 8.0), using a 10kDa MWCO membrane (Millipore, #Cat UFC8010) for 48hours, changing dialysate every 12 hours. Proteins were concentrated ultrafiltration using a 10kDa MWCO membrane (Millipore, cat#: UFC903024) and purified by IMAC (HisTrap HP, GE Healthcare), then SEC (Superdex 75, GE Healthcare). Monodisperse fractions of appropriate molecular weight were concentrated and exchanged by ultrafiltration using a 4kDa membrane (Millipore, Cat#: UFC8030) into HBS (HEPES 10mM, NaCl 150mM), then snap-frozen in liquid nitrogen and stored at −80°C until use.

### Protein crystallization and Data Collection

TCR 47BE7 and H2-D^b^/_68_ YGFRNVVHI were refolded and purified separately as described above. Protein crystallization was performed by sitting drop vapor diffusion. 96-well Intelli-plates (Art Robbins Instruments) were seeded with a Mosquito crystallization Robot (SPT Labtech) utilizing a 1:1 v/v protein to precipitant ratio then incubated at 18°C until crystal formation. H2-D^b^/YGFRNVVHI formed prism-shaped crystals (Tris-HCl 0.1 M, pH 8.5, sodium acetate 0.2 M, PEG3350 20–25%). Crystals were cryoprotected with the same mother liquor, supplemented with ethylene glycol 25% v/v then flash-frozen in liquid nitrogen and stored until use.

The ternary TCR 47BE7/H2-D^b^/_68_ YGFRNVVHI complex was formed by mixing both proteins in a 1:1 molar ratio, concentrating the mixture to 8mg/mL by ultrafiltration using a 30 kDa MWCO membrane (Millipore, Cat#: Z717185). The ternary complex formed rod-shaped crystals (Bis-Tris 0.1 M, pH 5.5, PEG3350 20–30%, lithium sulfate 0.2 M, glycerol 15% v/v). Crystals were directly flash-frozen in liquid nitrogen and stored until use.

### Structure Solution and Refinement

X-ray diffraction data were collected at Argonne APS beamline 19BM. Data were indexed, integrated, and scaled using HKL3000 and the AIMLESS/CCP4 program suite. The crystal structure of H2-D^b^/YGFRNVVHI was solved using PHASER with the reference search model (PDB: 5OPI) and refined using REFMAC and COOT. The H2-D^b^/_68_ Y GFRNVVHI binary structure coordinates and structure factors are accessible via Protein Data Bank accession code (PDB: 7N9J). The structure of TCR 47BE7/H2-D^b^/_68_ YGFRNVVHI ternary complex was solved using the binary complex as a search model, the 2FoFc map of which was used to sequentially build and refine the TCRα and TCRβ chains for TCR_47BE7 heterodimer by alternating refinement in REFMAC with model building and refinement in COOT. The 47BE7/H-2-Db/_68_YGFRNVVHI ternary complex coordinates and structure factors are accessible via Protein Data Bank accession code (PDB: 7NA5)

### Structural Modeling

Structural modeling of wild-type H2-D^b^/Hsf2_68–76_ was performed using Rosetta. The high resolution crystal structure of H2-D^b^/Hsf2 p.K72N_68–76_ served as the template, into which the position 5 arginine to lysine mutation was introduced using the PyMol mutagenesis function. Energy minimization was then performed using FlexPepDock (59). The top scoring models were visually inspected in PyMol, Ca and side-chain positioning was determined to be similar. The top scoring model was selected for display. Images were generated using PyMol.

## Extended Data

**Extended Data Table 1:** X-ray Data Collection and Refinement Statistics

Parameter	pMHC-I PDB Id: 7N9J	TCR-MHC PDB Id: 7NA5
Space group	C12_1_1	C222_1_
Resolution range (Å)	44.10 – 1.74	50.1 – 2.50
Cell dimensions	Å=104.42 56.96 72.77 β=94.84°	Å=155.44 191.71 67.81 αβγ=90°
Data redundancy	3.8(3.4)	5.9(4.5)
Mean I/σ(I), signal to noise ratio	26.4(2.53)	10.9 (1.3)
CC_1/2_ (highest resolution bin)	0.85	0.41
Completeness of data(%)	98.7	99.3
R_merge_	0.049	0.161
Refinement, R/R_free_	0.165/0.213	0.184/0.249
R/R_free_ (highest resolution bin)	0.227/0.287	0.321/0.345
F_o_,F_c_ correlation	0.97	0.96
Total number of atoms	3657	6695
Average B factor, all atoms (Å^2^)	35.2	51.3
Ramachandran plot, most favored, %	97.22	94.15
Allowed, %	2.78	5.85
Disallowed, %	0.00	0.00
RMS deviations, bonds, Å	0.010	0.006
RMS deviations, angles, °	1.553	1.432

## Data Availability

The H-2-Db/hβ2M/_68_ YGFRNVVHI binary structure coordinates and structure factors are accessible via Protein Data Bank accession code (PDB: 7N9J) The H-2-Db/hβ2M/_68_YGFRNVVHI:47BE7 ternary complex coordinates and structure factors are accessible via Protein Data Bank accession code (PDB: 7NA5). Software used in this study is available online: Vaxrank: https://github.com/openvax/vaxrank.
